# Evolution of Life on Earth: tRNA, Aminoacyl-tRNA Synthetases and the Genetic Code

**DOI:** 10.3390/life10030021

**Published:** 2020-03-02

**Authors:** Lei Lei, Zachary F Burton

**Affiliations:** 1 Department of Biology, University of New England, Biddeford, ME 04005, USA; llei@une.edu; 2 Department of Biochemistry and Molecular Biology, Michigan State University, E. Lansing, MI 48824-1319, USA

**Keywords:** aminoacyl-tRNA synthetase, Elongation Factor-Tu latch, evolution, genetic code, last universal common cellular ancestor, origin of life, polyglycine, tRNA

## Abstract

Life on Earth and the genetic code evolved around tRNA and the tRNA anticodon. We posit that the genetic code initially evolved to synthesize polyglycine as a cross-linking agent to stabilize protocells. We posit that the initial amino acids to enter the code occupied larger sectors of the code that were then invaded by incoming amino acids. Displacements of amino acids follow selection rules. The code sectored from a glycine code to a four amino acid code to an eight amino acid code to an ~16 amino acid code to the standard 20 amino acid code with stops. The proposed patterns of code sectoring are now most apparent from patterns of aminoacyl-tRNA synthetase evolution. The Elongation Factor-Tu GTPase anticodon-codon latch that checks the accuracy of translation appears to have evolved at about the eight amino acid to ~16 amino acid stage. Before evolution of the EF-Tu latch, we posit that both the 1st and 3rd anticodon positions were wobble positions. The genetic code evolved via tRNA charging errors and via enzymatic modifications of amino acids joined to tRNAs, followed by tRNA and aminoacyl-tRNA synthetase differentiation. Fidelity mechanisms froze the code by inhibiting further innovation.

## 1. Introduction

**Figure 1:**
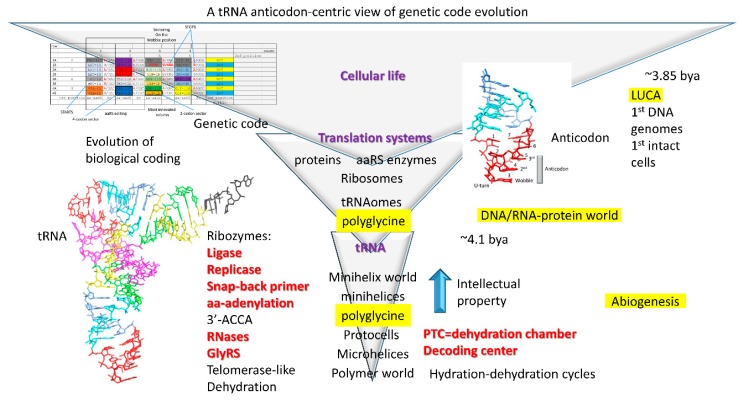
A model for evolution of life on Earth. Evolution of tRNA and translation systems leads to the first cells. A small number of ribozymes appears sufficient to generate tRNA from pre-tRNA sequences that are known because they are conserved in tRNAs. Red type indicates ribozymes generated in vitro. Evolution of tRNA leads to evolution of translation systems and the genetic code. Triangles indicate the increases in biological potential associated with advances in coding [1]. Abbreviations: PTC, peptidyl transferase center; LUCA, last universal common cellular ancestor. A version of this figure was published in [1] and is reprinted here with permission.

Figure 1 shows a schematic model for evolution of life on Earth [1]. Life evolved around tRNA and the tRNA anticodon. Notably, without tRNA, no complex life supported by genetic coding could evolve. Abiogenesis is the physical and chemical process by which pre-life led to cellular life. We refer to a late stage of abiogenesis, characterized by rapidly evolving translation systems, as the DNA/RNA-protein world. LUCA (the last universal common cellular ancestor) indicates the first cells and the first intact DNA genomes. We posit that tRNA was the essential biological intellectual property that drove evolution. We posit that the first tRNA was a tRNA^Gly^ charged by a ribozyme GlyRS (Glycine aminoacyl-tRNA synthetase) [1,2,3]. The initial purpose of tRNA was to generate polyglycine as a cross-linking agent to stabilize protocells. From tRNA arose tRNAomes (all of the tRNAs for an organism), ribosomes, the genetic code, and protein synthesis. Before tRNA, a minihelix world and a polymer world prevailed. Evidence for these more ancient worlds is conserved in tRNA sequences [4]. A dominant purpose of polymers and minihelices was also the synthesis of polyglycine, so initially tRNA evolved as an improved mechanism for polyglycine synthesis. Once the genetic code evolved, synthesis of RNA-encoded proteins was possible, and complex life on Earth became inevitable. Triangles in the figure indicate the expansions in biological capacity with innovations in coding, and Darwinian selections became more stringent with successive enhancements in biological information processing. In this report, we return to this figure as we add additional detail and support to the model.

## 2. This Report

The purpose of this report is to focus the broader origin-of-life field on its central story, which is evolution of tRNA and the genetic code. Once tRNA evolves, evolution of tRNAomes and the genetic code became inevitable. The evolution of genomes, cells, replication systems, and biological complexity then became inevitable. Recently, a review described the origin-of-life field partly in terms of conflicting approaches and philosophies [5]. In this report, we consider a lack of focus in origin studies to be an issue. Another recent paper made a bold attempt to describe the advent of biological coding [6], which we also consider.

The basic approach advocated here is to start at the center of a system of coevolutionary partners and to work out. tRNA is the central feature of the evolution of life (Figure 1). tRNAomes, the genetic code, mRNA, and ribosomes evolved around tRNA [1,7]. A strategy of beginning with the core was previously applied by us to analyze the evolution of transcription systems, including RNA polymerase, general transcription factors, and promoters [8,9,10]. Although transcription was then a more familiar problem to us, transcription systems are more derived evolutionarily than translation systems, making translation systems in some ways a simpler problem. By applying lessons from transcription systems, we rapidly gained insight into translation systems. We found that focus on tRNA was the key to understanding translation. Focusing on the central issue was also the strategy underlying the RNA world hypothesis, although we find the hypothesis is enriched by focus on tRNA. This report, therefore, focuses the RNA world approach on generation of tRNA and the genetic code. RNA, of course, and tRNA form the core of the genetic coding system. From RNA, DNA can be generated via reverse transcription. From RNA, protein can be generated via translation. As coding of protein enzymes evolved, in order to supply needed enzymes, ribozymes can function as catalysts. Here, we advocate a very similar approach to that underlying the RNA world hypothesis. The advantage of the inside-out approach is that we provide a means of thinking critically and holistically about ancient evolution problems, and we make potential sense out of otherwise bewildering complexity.

Investigators have advocated top-down and bottom-up approaches to analysis of ancient evolutionary events [5,11,12]. Here, we start with a top-down strategy of inferring pre-life from molecules such as tRNA that were conserved in the pre-life→cellular life transition. Studying tRNA evolution, the tRNA molecule suggests both top-down and bottom-up strategies to understand evolution of repeating polymers, inverted repeats, minihelices, tRNA, tRNAomes, ribosomes, and the genetic code (Figure 1) [1,7]. Significantly, tRNA was generated from highly patterned sequences: repeats and inverted repeats [1,3,4,13]. Therefore, tRNA indicates features of a minihelix world and polymer world that preceded tRNA. Understanding tRNA sequence and structure from existing sequences helps to model how tRNAomes and the genetic code evolved. Centering the focus on tRNA, ribosome evolution becomes easier to understand [7]. Analysis of tRNA, therefore, suggests both top-down and bottom-up strategies to model and understand the central story in evolution of life on Earth.

## 3. A Working Model for Evolution of the Genetic Code

### 3.1. Anticodon Preference Rules Govern Filling the Genetic Code

Amino acids appear to enter the genetic code according to ordered rules for tRNA anticodon preference. In the 2nd and 3rd anticodon positions, the preference appears to be C > G > U >> A [1]. Therefore, 2nd and 3rd anticodon position C is favored. We posit that C is favored in the anticodon because it is a pyrimidine (a smaller base), and C forms three hydrogen bonds to G in mRNA. G and U are approximately equally favored, but amino acids with anticodon 3rd position G enter the code before amino acids with 3rd position U, indicating a preference for anticodon G over U (see below). The explanation we offer is that G forms three hydrogen bonds, but is a purine (a larger base), and U forms two hydrogen bonds but is a pyrimidine (a smaller base). A is strongly disfavored, particularly in the 3rd anticodon position. A is disfavored because it forms two hydrogen bonds and is a purine (a larger base). Of course, despite preferences, single base recognition (A, G, C, and U) was essential in the 2nd and 3rd anticodon positions to evolve a complex code. The preferences we identify for tRNA anticodons could potentially be preferences for the complementary bases in mRNA. Perhaps insight could be obtained from molecular dynamics or quantum mechanical simulations of codon-anticodon pairs on the ribosome to help judge the precise chemistry underlying these base selections.

Preferences in the anticodon wobble position appear to be G > (U/C) >>>> A. Only purine versus pyrimidine resolution is achieved for the tRNA wobble position, limiting the size of the code [1]. This is why the genetic code is divided into mostly 4-codon sectors and 2-codon sectors, with few 1-codon sectors. In the wobble position, anticodon G reads mRNA codon C, as a Watson–Crick pair, and U, as a wobble pair. Anticodon U reads codon A, as a Watson–Crick pair, and G, as a wobble pair. Without modification, anticodon C reads mRNA codon G with reasonable fidelity. In evolution, anticodon wobble U and C are rarely or never split in coding two amino acids, as we discuss below. Anticodon wobble A is essentially never used in Archaea and Bacteria [14,15]. When A is modified to inosine, however, A may be encoded in tRNA in Bacteria and Eukarya.

### 3.2. Guidelines for Placements of Amino Acids into the Code

A highly detailed description of genetic code evolution is provided in this report. The working model is built on a simple set of ideas: (1) glycine was the first encoded amino acid, and at an early stage, all anticodons and all codons encoded glycine; (2) amino acids were added to the code via invasion of occupied sectors; (3) the genetic code evolved around the tRNA anticodon; (4) the final sectoring structure of the code depended strongly on the order of additions of amino acids; and (5) the code was highly structured according to its evolutionary history, and code structure was largely maintained through subsequent evolution. That glycine was the first encoded amino acid has also been hypothesized by others [16,17,18]. We posit this idea mostly based on the observation that tRNA^Pri^ (a primordial tRNA) is closest in sequence to tRNA^Gly^ in ancient Archaea [1,3]. Also, Gly occupies what appears to be the most favored anticodon positions in the code (tRNA^Gly^ (GCC, UCC, CCC); favored column 4 (2nd anticodon position C); favored row 4 (3rd anticodon position C)).

We posit that Gly, Asp, Glu, Ala, and Val are among the first encoded amino acids. All of these amino acids are found in favored row 4 of the code (3rd anticodon position C). Furthermore, Phe, Tyr, Trp, and Cys appear to be among the last amino acids encoded (disfavored 3rd anticodon position A) [19,20]. Also, stop codons are recognized in mRNA by protein translation release factors [21], so stop codons do not follow rules for tRNA anticodons. Phe, Tyr, Trp, Cys, and stop codons are all located in row 1 of the genetic code, indicating that row 1 (3rd anticodon position A) is disfavored. We conclude that the position of amino acids in the code reflects a Darwinian tRNA anticodon preference selection and also the order of amino acid additions to the code. The genetic code is highly structured according to its evolutionary history, and structure is demonstrated best by evolution of aminoacyl-tRNA synthetases (aaRS; i.e., GlyRS) [1,14]. We discuss the evidence below.

### 3.3. Darwinian Selection in a Pre-life World

There has been controversy about whether Darwinian selection can function before living systems evolve [5,6,11,12]. For instance, some have argued against evolution of the genetic code based on Darwinian selection. In this paper, we posit Darwinian selections for minihelices, tRNA, tRNAomes, ribosomes, and the genetic code. As indicated in Figure 1, we do recognize that Darwinian selections become more stringent as biological coding evolved. To us, there is no mystery about how to evolve the most central pre-life systems.

### 3.4. The Archaeal Domain is the Most Ancient

Although this point has been argued, we posit that, for translation functions, the archaeal domain is the most similar to LUCA and to pre-life [1,3,14,22]. We have previously argued this point based on evolution of transcription systems [8,9,10]. In compelling support of this hypothesis, archaeal tRNAs are more ancient than bacterial tRNAs [1,3,4]. This point can be demonstrated by inspection of archaeal and bacterial tRNAomes. Archaeal tRNAs are much closer to tRNA^Pri^ (a primordial tRNA). Bacterial tRNAs are more derived. Therefore, to understand evolution of the genetic code, we focus first on archaeal systems before determining how the genetic code differs in the bacterial system. Eukarya are a more complex problem, because eukaryotes arose as an archaeal and bacterial fusion.

### 3.5. Evolution of tRNA

Because life evolved around tRNA, solving the evolution of tRNA is a core issue in the evolution of life [1,2,3,4,13]. Fortunately, the evolution of tRNA is a simple problem. tRNA arose from ordered sequences: repeats (GCG, CGC and UAGCC) and inverted repeats (~CCGGGUUAAAAACCCGG). Despite some controversy [23,24,25], the evolution of tRNA is a known and solved problem [4]. Specifically, tRNA evolved from ligation of three 31-nt minihelices followed by 9-nt internal deletion(s). A single internal 9-nt deletion generated a type II tRNA (initially 84-nt) with an expanded variable loop. Two internal 9-nt deletions generated a type I tRNA (initially 75-nt). There are other opinions about tRNA evolution [23,24,25,26,27,28,29,30] that are inconsistent with tRNA sequences [4]. The three minihelix model for tRNA evolution, by contrast, fully describes genesis of tRNA from the beginning of the 5’-acceptor stem to the end of the 3’-acceptor stem [4]. The 3’-ACCA (initial sequence), to which the amino acid is bound, may have attached by ligation. The evolution of tRNA was solved as a puzzle, and this is a puzzle that anyone can solve. Because tRNA evolved from minihelices of known sequence, information is obtained about a minihelix world and a polymer world that preceded tRNA (Figure 1). 

### 3.6. The Minihelix and Polymer Worlds

We posit that polyglycine was generated in a pre-life world, as a cross-linking agent to stabilize protocells. Polyglycine (i.e., Gly_5_) is a component of bacterial peptidoglycan cell walls [31,32,33]. Gly_5_ is a cross-linker connecting short peptide chains (i.e., L-Ala-D-Glu-L-Lys-D-Ala) that are linked to glycan chains (i.e., (N-acetylglucosamine-N-acetylmuramic acid)_n_). Gly_5_ cross-links L-Lys on one peptide chain to D-Ala on another. We posit that, in the pre-life world, an analogous peptidoglycan coat was synthesized using ribozyme catalysts and assembled on protocells. One prediction of our model is that peptidoglycan coats enhance the functions of protocells for pre-biotic chemistry.

Because aspects of the more ancient minihelix and polymer worlds are known from current tRNA sequences [1,3,4,13], what were the functions of these ancient repeats and inverted repeats? We posit that a large set of 31-nt minihelices was utilized with RNA templates of diverse sequences to synthesize polyglycine, which stabilizes cells or protocells as a component of peptidoglycan [31,32,33]. In tRNA sequences, only two minihelices survive: a D loop minihelix (GCGGCGGUAGCCUAGCCUAGCCUACCGCCGC) and two copies of an anticodon/T loop minihelix (~GCGGCGGCCGGGUUAAAAACCCGGCCGCCGC). The only sequence ambiguity in the anticodon and T loop minihelices is in the ~UUAAAAA loops. There is no ambiguity in the 5’- (GCGGCGG) and 3’-acceptor stems (CCGCCGC) or in the 5-nt stems (5’ CCGGG and 3’ CCCGG). We posit that minihelices evolved to synthesize polyglycine using mixed mRNA templates, and mixed 31-nt minihelices, including some no longer known. A D loop minihelix would be expected to project a single C to recognize G in a mRNA (1-nt code). An anticodon/T loop minihelix would be expected to project ~AAA to recognize ~UUU in a mRNA (3-nt code). Although only two minihelix sequences survived in tRNAs, it is expected that many minihelices with different core sequences than those described here helped a primitive translation system to recognize different mRNA sequences. In order to attach glycine using a ribozyme GlyRS (GlyRS-RBZ), we posit that ACCA was ligated to 31-nt minihelices. A primitive decoding center scaffold and a mobile peptidyl transferase center (PTC) appear sufficient to serve as an ancient ribosome [7].

Because minihelices were generated from GCG, CGC, and UAGCC repeating polymers, a mechanism must have existed to generate these repeats. Although there may be other possibilities, we posit that RNA repeats may have been synthesized via a telomerase-like ribozyme with a guide RNA template. These and other RNAs may have been replicated via a template-dependent ribozyme replicase (Figure 1). A mechanism must have existed to generate RNA fragments of defined lengths (i.e., 7-nt (acceptor stems) and 17-nt (minihelix cores)). To attach fragments of RNA, a ribozyme ligase was required. A ribozyme replicase must have existed to utilize and replicate inverted repeats. A 31-nt minihelix ligated to an RNA can be utilized as a snap-back primer for replication of the complementary strand. A ribozyme GlyRS must have existed, but no other aminoacyl transferases were initially required. The system requires RNA scaffolds to act as a primitive decoding center. The system further requires a mobile peptidyl-transferase center [34]. We posit that the primitive ribosome was approximately built on this model [7]. Such an ancient minihelix world could be reconstructed in a laboratory and/or generated computationally to challenge these ideas. All of the requisite ribozymes have been generated or approximated through selection in vitro [34,35,36,37,38,39,40,41,42,43,44]. Although not discussed here, similar hypotheses can be posited for the more ancient polymer world. It was initially a surprise to us that the central functions of life appear to have been generated from ordered, rather than random polymer sequences. Existing tRNA sequences show that life evolved, at least in part, from ordered sequences (repeats and inverted repeats).

So, we posit that the Darwinian selection for the minihelix world was primarily to synthesize polyglycine to stabilize protocells (Figure 1). Protocells were necessary to generate membrane potentials to harness redox energy. Initially, tRNA evolved from minihelices as an improved mechanism to generate polyglycine. Evolution of tRNA eventually established the 3-nt genetic code. From this modest beginning, the genetic code evolved, driven by Darwinian selection.

### 3.7. The Genetic Code Has Order

A number of models have been advanced to describe the evolution of the genetic code [6,28,45,46,47,48,49,50,51,52]. With the exception of our model, we find these alternate opinions to be potentially flawed. Specifically, alternate models show the genetic code in terms of mRNA codons, rather than tRNA anticodons. As we have shown, however, the genetic code complexity was determined by how the tRNA anticodon was read on the ribosome. Reading of the tRNA anticodon, therefore, limited the size and complexity of the genetic code [1,3,13,14]. Sixty-four codons are recognized in mRNA (4 × 4 × 4), but, in tRNA, the maximal complexity of the genetic code is 32 anticodons (2 × 4 × 4), explaining why only 20 amino acids and stop codons were encoded. Most notably, only pyrimidine versus purine discrimination was initially permitted at the anticodon wobble position, limiting the size of the code in tRNA. 

**Figure 2:**
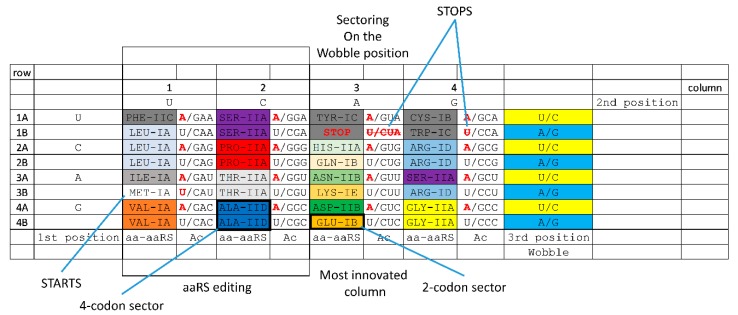
The genetic code is highly structured. A 32-assignment codon-anticodon (Ac) table is shown. Codon sequences are shown on the outside (1st position, 2nd position, and 3rd wobble position). aaRS enzymes are indicated by their structural subclass (i.e., GlyRS-IIA). aaRS enzymes that edit inaccurately attached amino acids (aa) are found in columns 1 and 2. The color shading scheme reflects how amino acids were added to the code and is described in future figures. Red letters indicate very rarely used tRNAs and stop codons (strike-through) [1].

**Figure 3:**
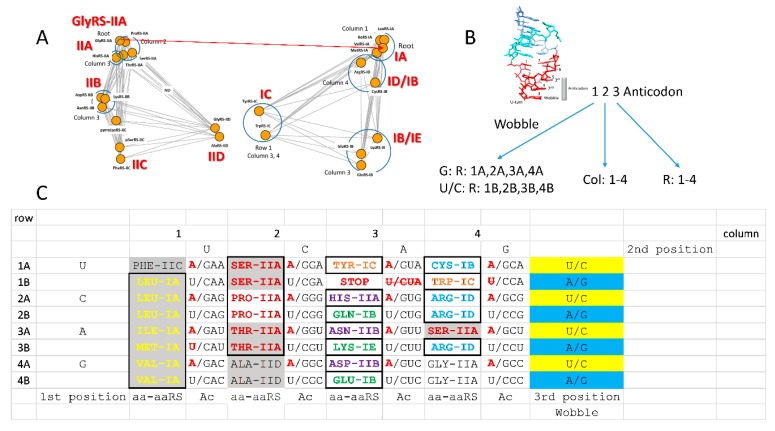
Evolution of the genetic code occurred mostly in columns. (**A**) Evolution of aaRS enzymes. Distances represent evolutionary differences. The red arrow indicates that ValRS-IA is derived from its sequence homolog GlyRS-IIA. (**B**) The relationship of the tRNA anticodon to the genetic code columns (Col) and rows (R). (**C**) The genetic code in Archaea. Grey shading indicates aaRS that possess a separate active site to edit inappropriately attached amino acids [56]. Colors highlight genetically similar aaRS enzymes demonstrating evolution primarily in columns. A version of this figure was previously published in [1] and is reprinted here with permission.

Because tRNA limits the size of the genetic code, we show the code as a 32-assignment, codon-anticodon table (Figure 2 and Figure 3). Figure 2 is annotated to describe important features of the code (i.e., start and stop codons). Because of the history of code evolution, the genetic code is primarily broken into 2-codon and 4-codon sectors. Because of tRNA anticodon wobble ambiguity, separating 1-codon sectors to encode two amino acids poses a challenge. Trp (CCA) is a special case because stop codon (UGA; anticodon UCA) is recognized only as a codon in mRNA by a protein translation release factor [21], which does not obey tRNA anticodon rules. In Archaea, Ile (CAU) and Met (CAU) anticodons co-occupy a sector and are discriminated by distinct anticodon wobble C modifications [53,54,55]. In order to reduce translation errors, tRNA^Ile^ (UUA) anticodons are rarely used in prokaryotes. Ile (CAU) and Met (CAU) anticodons provide insight into how amino acids invaded the code. Therefore, apparent 1-codon sectors are special cases that can be ignored in the initial establishment of the code. Because the code does not divide easily into 1-codon sectors, the initial maximal code complexity must be 32-assignments as shown in Figure 2 and Figure 3.

### 3.8. History of Genetic Code Evolution is Largely Preserved in aaRS Evolution Patterns

Aminoacyl-tRNA synthetases (aaRS; i.e., GlyRS) belong to two structural classes (class I and class II; i.e., GlyRS-IIA) with many structural subclasses (A–E) (Figure 2 and Figure 3). Class I and class II aaRS have incompatible folds. Sometimes, structural subclasses do not adequately reflect evolutionary relatedness of aaRS enzymes, but these relationships have recently been clarified (Figure 3A) [1]. Class II aaRS are older than class I aaRS. GlyRS-IIA is the primordial aaRS from which all aaRS enzymes were derived. As we have shown, GlyRS-IIA is a simple sequence homolog of ValRS-IA and IleRS-IA [1,14]. GlyRS-IIA was refolded into (probably) ValRS-IA from which other class I aaRS enzymes radiated [1]. Much of the structure of the genetic code and the history of genetic code evolution can be inferred from the relatedness of aaRS enzymes. The distributions of related aaRS enzymes indicate that much of the evolution of the genetic code occurred within code columns (Figure 3C). Code columns relate to the middle position of the tRNA anticodon, which is the most important position for translational accuracy (Figure 3B). Because the genetic code has a highly conserved structure, a history of genetic code evolution is preserved, particularly in aaRS evolution patterns.

### 3.9. The “Frozen Accident”

The genetic code is highly structured, indicating that a history of its evolution is recorded and preserved (Figure 2 and Figure 3). Conservation of structure in the code is reminiscent of Francis Crick’s “frozen accident” [1,45,46,57,58,59], which we interpret as a rapid evolution to an enduring form with very little subsequent movement or replacement of amino acids within the code. Our approach to describe the sectoring of the code has been explained in some detail, but in this report, we improve and extend our previous model. Notably, making a small number of simplifying assumptions, the entire sectoring of the code can be described via Darwinian selection. Although we relate events from ~4 billion years ago, we consider these detailed working models to be very useful to strengthen our understanding of the evolution of the code.

Establishment of the genetic code requires consideration of a challenging “chicken and egg” problem: that is, how do you establish the functions and structural sub-classes of aaRS enzymes before the code evolves to provide complex proteins [1,12]? We posit that this is part of the frozen accident. We posit that the code was sectored initially by ribozyme aaRS enzymes. Once the code was sufficiently established, protein aaRS enzymes replaced their ribozyme analogues. aaRS enzyme divergence was driven by the pressures caused by the increasing requirements for accurate coding. Such a model requires very rapid and almost irreversible establishment of the genetic code, consistent with conserved code structure (Figure 2 and Figure 3). We reiterate that there is very little evidence for migration of amino acids in the code. Ser and Arg may be exceptions.

Analysis of tRNAomes indicates much higher order in more ancient species such as ancient Archaea [2]. By contrast, bacterial tRNAomes are much more derived. We posit, therefore, that tRNAomes are highly dynamic in evolution, and that much of the initial patterning of tRNAomes has been lost, as tRNAs mutate, in order for, for instance, aaRS enzymes to distinguish one tRNA from another. tRNAomes occupy a limited evolutionary space. Because tRNAs are small RNAs that must maintain their characteristic structure, tRNAomes undergo both divergent and convergent evolution within a constrained Darwinian space. Therefore, the frozen accident, which was initially established in the tRNAome, is now more apparent in aaRS sequences and structures than in tRNAomes. We posit that during genetic code sectoring, tRNAomes were more highly structured, and that, when proteins gained sufficient complexity, aaRS evolution tracked the highly ordered tRNAome. After sectoring of the code and aaRS enzymes was mostly complete, Darwinian selection drove diversification of tRNAomes to enhance translational accuracy. Consistent with this view, we observe that archaeal tRNAomes are more highly structured (and more similar to LUCA) than bacterial tRNAomes, which are more derived [3]. We posit that the ~8→~16 amino acid transition (see below) provides proteins of sufficient complexity to evolve the patterned code.

The tRNAome is an enclosed evolutionary space because tRNAs must maintain their shape and structure. Analysis of tRNAomes indicates both convergent and divergent evolution of tRNAomes. For instance, for the most part, the *Pyrococcus furiosis* (an ancient Archaea) tRNAome resembles a highly ordered LUCA tRNAome. By contrast, *Escherichia coli* (a highly derived Bacterium) has a much more scrambled tRNAome with little apparent evolutionary order. As evidence of continuing tRNA evolution, we have presented evidence in Archaea for recruitment of a duplicated tRNA^Pro^ to become reassigned as a tRNA^Trp^ and a duplicated tRNA^Trp^ to become reassigned as a tRNA^Phe^, indicating how tRNAs diverge, converge, and jump within a tRNAome [3]. To avoid errors in coding, tRNAs sometimes jump to a neighboring column within a genetic code row, in contrast to aaRS enzymes that tend to evolve within columns (Figure 3). We posit that initially, tRNAomes mostly evolved within columns, followed by tRNAome divergence that includes tRNA jumping to a neighboring column but often maintaining the original row.

### 3.10. Polyglycine World

We posit that the initial selection to evolve the 3-nt genetic code was to synthesize polyglycine to stabilize protocells (Figure 1). Polyglycine (i.e., Gly_5_) is a cross-linking agent in the peptidoglycan layer of bacterial cell walls [31,32,33]. We posit that, before the advent of true cells at LUCA, protocells may have been stabilized by a very similar peptidoglycan structure: i.e., glycan chains with short polypeptide linkages (i.e., added by ribozymes) including polyglycine cross-links. Such a scenario makes polyglycine of selectable value in the primordial world. We posit that the prebiotic world generated peptidoglycan supports for protocells. This hypothesis suggests that, for prebiotic chemistry, protocells with a peptidoglycan cage would have advantages over naked protocells. Others have also proposed that glycine was the first encoded amino acid [16,17,18]. So far as we know, we are the first to propose that the initial entire genetic code (all anticodons and all codons) encoded polyglycine. This simple assumption enables a detailed model for evolution of life on Earth. 

### 3.11. Evolution of the Genetic Code (A Working Model)

We posit that the genetic code initially evolved primarily to synthesize polyglycine used as a cross-linking agent to stabilize protocells (Figure 1). What this indicates is that, after evolution of tRNA, the tRNA anticodon mutated rapidly to all possible sequences. We imagine a primordial world in which tRNA can replicate and mutate rapidly. tRNA sequences other than the anticodon posed difficulties to mutate because alterations in stems or in the “elbow” (where the D loop, V loop, and T loop interact; the bend of the tRNA “L” shape) likely require compensatory mutations to stabilize the tRNA fold. After generating many anticodon mutations, all anticodons and all mRNA codons encoded glycine to synthesize polyglycine. We posit that a GlyRS ribozyme charged these tRNAs (mostly) with glycine. Mistakes in aminoacyl-tRNA charging and modifications of amino acids linked to tRNAs drove the evolution of the code.

Because tRNA has a specialized 7-nt anticodon loop, the 3-nt tRNA anticodon projects to recognize a 3-nt mRNA codon. On a primitive ribosome, reading 3-nt codons, however, presented some difficulties that can still be recognized in wobble anticodon-codon interactions (Figure 3B). On the ribosome, the accuracy of the anticodon-codon interaction is monitored by the EF-Tu latch [60,61,62,63]. EF-Tu is a GTPase that enforces Watson–Crick geometry on the anticodon-codon interaction at the 2nd and 3rd anticodon positions. By contrast, the 1st anticodon wobble position is monitored by the latch, but, at the wobble position, only pyrimidine-purine recognition is initially achieved. The 2nd anticodon position is recognized with the highest accuracy. The 3rd anticodon position also requires Watson–Crick geometry. Rarely, in translation errors, G~U wobble pairs occur at the 3rd anticodon position, but G~U wobble pairs require tautomerization of either the anticodon or codon base to allow Watson–Crick geometry. Because of the EF-Tu latch, generally, unstable wobble pairs in the 3rd anticodon position are rejected before amino acid misincorporation. 

The genetic code evolved according to these tRNA anticodon recognition rules (2nd > 3rd > 1st (wobble) anticodon positions). To generate polyglycine, little translational accuracy in reading the anticodon is initially required because all anticodons encoded Gly. As amino acids invaded the code, we posit the following. The code generally sectored first around the 2nd anticodon position (most important), followed mostly by the 3rd position (next most important), followed by the wobble position (generally least important) [1]. Therefore, the genetic code evolved mostly around the tRNA anticodon, generating a clear working model for evolution of the code. In the model advocated here, the entire code is first populated with Gly. As other amino acids enter the code, Gly is displaced. As Gly is displaced, however, Gly retains the most-favored anticodons, based on a clear set of selection rules, and Gly gives up less favored anticodons to incoming amino acids [1]. According to such a model, the placements of all amino acids in the genetic code can be readily described. Alternate models for populating the genetic code are more problematic. Any model that attempts to populate the code from a single sector followed by migrating into other sectors appears unlikely [28,64,65]. For such an alternate model, how can a Darwinian selection be imagined for matching assignments of codons and anticodons? In our model, codon and anticodon specifications coevolve, and evolution of the tRNA anticodon drives evolution of codons. We find alternate models to make few useful predictions. By contrast, our model makes many predictions, many of which are justified. 

**Figure 4:**
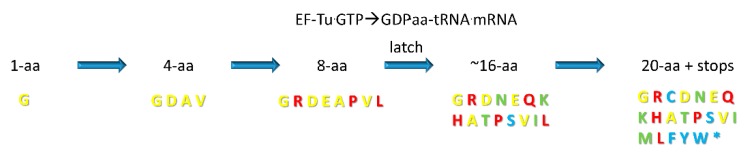
A proposed order of addition for amino acids (aa) to the genetic code. Amino acids appear to invade by genetic code rows. Yellow) Row 4 amino acids; Red) Row 2 amino acids; Green) Row 3 amino acids; Cyan) Row 1 amino acids and stop codons (asterisk). Leu, Ser and Arg are only scored with a single color. For simplicity, only a final, primary position in the code is scored by color.

In Figure 4, we show a proposed order of addition for amino acids into the genetic code. We posit that the genetic code evolved from a 1-amino acid code (Gly) to a 4-amino acid code (Val, Ala, Asp, Gly) to an 8-amino acid code (Val, Leu, Ala, Pro, Asp, Glu, Arg, Gly). The selection to add amino acids to the code was to generate novel polypeptide products. Sectoring to a 4-amino acid code required single-base recognition of the 2nd anticodon position. Evolution to an 8-amino acid code required recognition of two anticodon positions, but in the absence of the EF-Tu latch [60,61,62,63], recognition of the 1st wobble base and the 3rd anticodon position were both wobbly, and only pyrimidine versus purine recognition was initially achieved. Therefore, to advance beyond an 8-amino acid code required evolution of the EF-Tu latch to confer single-base recognition at the 2nd and 3rd anticodon positions. At the 8-amino acid stage, we posit that genetic code columns 1, 2, and 4 sectored by one mechanism, and column 3 sectored by a slightly different mechanism. Columns 1, 2, and 4 sectored according to the 3rd anticodon position (2nd + 3rd anticodon positions). Column 3 sectored according to the 1st (wobble) position (2nd + 1st anticodon positions). Essentially, in order to expand the genetic code, the primitive ribosome and translation system were “learning” (teaching themselves) to read just two out of three anticodon bases. The evolutionary history of column 3, sectoring on the 1st anticodon wobble base rather than the 3rd anticodon position, caused column 3 to become the most innovated column in the code.

For the code to continue to sector requires evolution of the EF-Tu GTPase anticodon-codon latch (Figure 4). From the 8-amino acid code, we posit that the code sectored to approximately a 16-amino acid code (i.e., ~Gly, Arg, Asp, Asn, Glu, Gln, Lys, His, Ala, Thr, Pro, Ser, Val, Ile, and Leu). This transition requires evolution of the EF-Tu latch because the 2nd and 3rd anticodon positions must be read with single-base resolution to achieve an ~16-amino acid code, and in the absence of the latch, the 3rd anticodon position is read essentially as a wobble position, with more difficulty than the 2nd position. At the ~16 amino acid stage of evolution, additional amino acids can be added, but as the system evolves enhanced translational accuracy, fidelity restrictions create an ever-more stringent barrier to encoding additional amino acids. The reason for this limitation is that amino acids are added to the code through mischarging of tRNAs followed by selection for a more complex and innovated code. Therefore, addition of amino acids to the code is positively selected, because a richer code makes encoded proteins more complex, but negatively selected because additions increase tRNA-charging and translation errors, forcing additional challenges to the system. Potentially, a 32-assignment code could have evolved (i.e., 29 amino acids + 3 stop codons). We posit that some sectors of the code are difficult to split, however, from 4-codon sectors to 2-codon sectors because these divisions cause error catastrophe, particularly for the left half of the genetic code. Hydrophobic and neutral amino acids encoded by the left half of the code have little amino acid side chain character for recognition by aaRS enzymes, so splitting a 4-codon sector into two 2-codon sectors might cause too frequent tRNA charging errors [1,14].

### 3.12. Evolution of the Genetic Code within Columns

**Figure 5:**
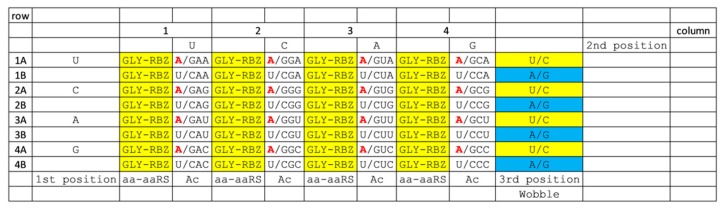
The 1-amino acid code. All tRNAs are tRNA^Gly^. aaRS designations are indicated, although, through multiple steps, ribozyme (RBZ) aaRS enzymes were initially probably responsible for aminoacylating tRNAs. Ac) anticodon.

**Figure 6:**
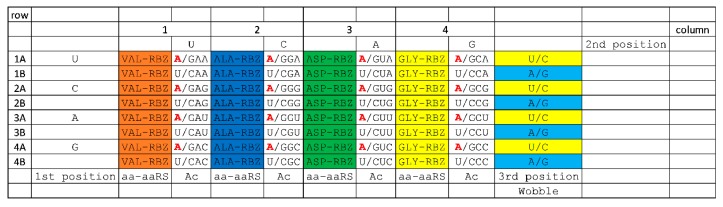
The 4-amino acid code. Colors follow amino acids through code sectoring. Bases indicated in red type are disallowed (wobble A is very rare in Archaea) [1,14].

**Figure 7:**
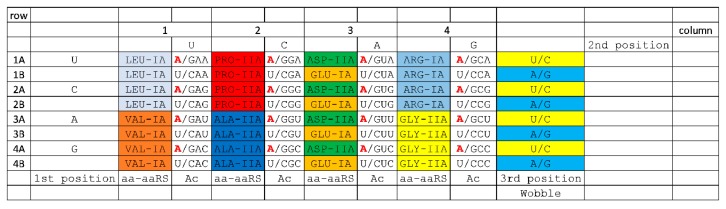
The 8-amino acid code. Columns 1, 2, and 4 sector on the 2nd and 3rd anticodon positions. Column 3 sectors on the 2nd and 1st (wobble) anticodon positions, leading to column 3 complexity.

**Figure 8:**
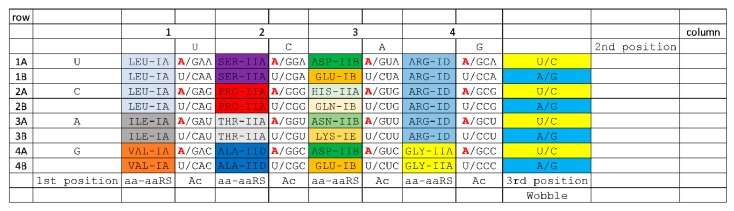
The ~16-amino acid code after evolution of the EF-Tu latch. At this stage, proteins may take on sufficient complexity to replace ribozyme aaRS enzymes.

**Figure 9:**
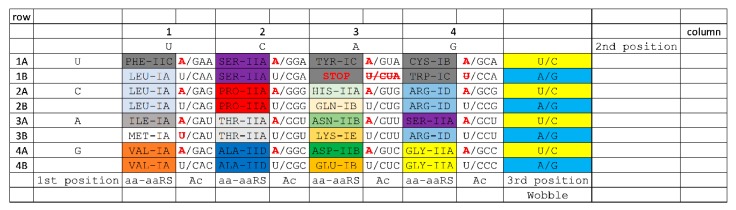
The standard 21-assignment genetic code (20-amino acids + stops) in Archaea. Amino acids and stop codons shaded in charcoal were late additions to the code (row 1). Column 1, row 3B (anticodon CAU) is co-occupied with Ile-IA and Met-IA.

Because genetic code columns appear to dominate evolution of the code (Figure 2 and Figure 3), how can this be explained? We posit that evolution of the genetic code is dominated by the tRNA anticodon. The second anticodon position, which is most important for translational accuracy, is represented by genetic code columns (Figure 3B). Therefore, the genetic code evolved primarily within columns, rather than within rows, or according to a random distribution. Based on this reasoning, a simple, step-by-step model for genetic code evolution is proposed in Figure 5, Figure 6, Figure 7, Figure 8 and Figure 9. Figure 5 shows the initial evolution of the code to encode polyglycine. Figure 6 shows the 4-amino acid code to encode Gly, Asp, Ala, and Val [1,6,46,47]. Figure 7 shows wobbly sectoring to an 8-amino acid code. Figure 8 shows evolution to a ~16-amino acid code, after evolution of the EF-Tu latch [60,61,62,63]. Figure 9 shows evolution to the standard genetic code (21 assignments: 20-amino acids + stops). 

### 3.13. Column 2

Column 2 of the genetic code sectored evenly into four 4-codon sectors encoding Ala, Thr, Pro, and Ser (Figure 8 and Figure 9). Because of early wobbly recognition of the 3rd anticodon position, however, we posit that column 2 may have initially sectored into two 8-codon sectors encoding Ala and Pro (Figure 7). Essentially, the idea is that, as the ribosome evolves to read the 3rd anticodon position, initially only pyrimidine versus purine discrimination was possible. Only after evolution of the EF-Tu anticodon-codon latch, is single base recognition at the 3rd anticodon position achieved (Figure 8). We posit that Ala, which we posit entered the code before Pro, protected the more favored sectors, according to the 2nd and 3rd position anticodon rules C > G > U >> A. Because the 4th row of the code is favored (3rd position C), Ala protected favored sectors from invasion by Pro. After evolution of the EF-Tu latch, the entire anticodon could be read on the ribosome. Thus, Thr could invade the Ala sector with Ala retaining the most favored rows (row 4; 3rd anticodon position C). Ser could invade the Pro sector. Amino acids that entered the code first, therefore, retained the most favored rows in the code. Because column 2 has no 2-codon sectors, column 2 has no sectoring to discriminate amino acids according to the wobble position. In part, we posit that column 2 amino acids (Ala, Thr, Pro, and Ser) have too little character to be more accurately distinguished within their aaRS active sites, limiting further innovation in column 2 from 4-codon sectors to 2-codon sectors [1,14]. Interestingly, ThrRS-IIA, ProRS-IIA, and SerRS-IIA are closely related aaRS enzymes by structure and sequence (Figure 3). The similarity of these enzymes indicates evolution within genetic code column 2. 

### 3.14. Columns 1 and 4

Columns 1 and 4 are posited to be more chaotic versions of sectoring, similar to the sectoring of column 2. Column 1 is posited to have sectored first between Val (8-codon sector) and Leu (8-codon sector) (Figure 7). Evolution of the EF-Tu anticodon-codon latch was necessary to sector further. Val was invaded by Ile, with Val retaining the most favored sectors (row 4; 3rd anticodon position C). We posit that, prior to Met invasion, there were three tRNA^Ile^ anticodons (GAU, UAU, and CAU) (in Archaea, AAU is rarely or never used). Ile (CAU) was invaded by Met (CAU). To retain Met in the code, Ile (UAU) was selected against, because tRNA^Ile^ (UAU) potentially reads both Ile (AUA) and Met (AUG) codons. Different modification enzymes evolved to alter the wobble base of tRNA^Ile^ (CAU) and tRNA^Met^ (CAU) so these tRNAs can be accurately discriminated, specifically reading Ile (AUA) and Met (AUG) codons. At an early time in evolution (i.e., before LUCA), therefore, two tRNA^Met^ (CAU), 1 elongator tRNA^Met^ (CAU) and 1 initiator tRNA^Met^ (CAU), and two tRNA^Ile^ (GAU and CAU) were utilized [53,54]. There are minor alterations to this common situation in some organisms. In eukaryotes, more tRNA^Ile^ anticodons are utilized (UAU and IAU; I for inosine (adenine→inosine)), and additional rules apply [14]. Phe was a late addition to the code invading a Leu sector. As a late invader, Phe is relegated to disfavored row 1 (3rd anticodon position A). In previous publications, our laboratory has somewhat confused discussion of Ile and Met sectoring. We apologize. Ile and Met sectoring is a fundamental but simple story in structuring of the code. Essentially, Met invaded a 4-codon Ile sector, but the invasion was never fully resolved and now can never be.

Interestingly, in column 1, Val, Ile, and Leu are hydrophobic amino acids. Furthermore, ValRS-IA, IleRS-IA, MetRS-IA, and LeuRS-IA are closely related enzymes by sequence and structure (Figure 3). We posit that amino acid hydrophobicity and conserved aaRS enzymes demonstrate evolution of the genetic code within column 1. We posit, further, that evolution of the genetic code within columns relates to the central importance of tRNA anticodon position 2. Met is in some ways a special case because Met is a late addition to the code and Met utilizes two tRNA^Met^ (CAU) at the base of code evolution (1 elongator and 1 initiator tRNA^Met^). Phe is also a late entry into the code. Phe is an aromatic and hydrophobic amino acid. Interestingly, PheRS-IIC helps to discriminate charging of tRNA^Phe^ (GAU) from charging by the class IA enzymes for other column 1 amino acids. All of the aaRS enzymes in column 1 have an editing active site to enhance the accuracy of tRNA charging [56]. Editing is posited to be important for these aaRS enzymes because hydrophobic amino acids have few discriminating features within the aaRS active site for accurate charging to tRNA. To protect against charging errors, column 1 has 4-codon sectors for Val and Leu. Ile was reduced to a 3-codon sector because of invasion by Met. Met (CAU) and Ile (CAU) share a 1 codon sector, but Met is required for translation starts, and Met was a late addition to the code. Although Met invading the Ile sector was never fully resolved during code establishment, Met invading the Ile sector suggests a mechanism for the previous successful invasions of other sectors of the code by other amino acids.

Column 4 may have sectored as follows. Initially, column 4 split between Gly and Arg (8-codon sectors), with Gly occupying the favored rows 3 and 4 (Figure 7). With evolution of the anticodon-codon EF-Tu latch, Arg invaded row 3, displacing Gly. We posit that Ser invaded column 4 to attain a better anticodon with 2nd anticodon position C (column 4; anticodon GCU). Ser was able to invade column 4 because tRNA^Ser^ is a type II tRNA with an expanded variable loop, which is recognized as a determinant by SerRS-IIA for accurate amino acid placement [56]. Other tRNAs generally depend strongly on recognition of their anticodon, for amino acid placement by their aaRS, making such a jump within the genetic code impossible, without inducing error catastrophe. The Arg sector could be invaded by Ser because ArgRS-ID cannot charge type II tRNA^Ser^ (GCU) with Arg. Ser is the only amino acid that is split between columns (in this case columns 2 and 4) within the genetic code table. We posit that Ser is the only amino acid to have jumped successfully in evolution of the code, and that only SerRS-IIA and type II tRNA^Ser^ could readily support such a jump. Because ArgRS-ID is a class I aaRS, invasion of the Arg sector by SerRS-IIA may have been facilitated. In column 4, Trp, Cys and a stop codon are late entries into the genetic code that are relegated to disfavored row 1 (3rd anticodon position A). Stop codons are recognized by proteins, not tRNA [21], so stop codons do not follow tRNA anticodon selection rules. Aromatic amino acids Phe (column 1), Tyr (column 3), and Trp (column 4) are late entries into the genetic code, all found in disfavored row 1 (3rd anticodon position A).

### 3.15. Column 3 

Column 3 is the most innovated column in the code. We posit that the reason for such high innovation is because column 3 sectored initially on the 2nd and 1st (wobble) anticodon positions (Figure 7). Columns 1, 2, and 4, by contrast, sectored initially on the 2nd and 3rd anticodon positions, as described above. We posit that column 3 initially sectored between Asp and Glu, with Asp utilizing 1st wobble anticodon position G, and Glu utilizing 1st wobble anticodon position U/C, creating the striped pattern. Because we posit that Asp entered the code before Glu, we posit that a preference is indicated for selection of wobble anticodon position G over U/C. A possible reason for this preference is that only one tRNA (GUN) is necessary to recognize two mRNA codons, rather than two tRNAs (UUN, CUN). In the wobble position, G pairs with C as a Watson–Crick pair, and G forms a wobble pair with U. Full establishment of the three-nucleotide code required the EF-Tu latch. Once the latch evolved, other amino acids could invade column 3. Initially, Asp is posited to have occupied all sectors in column 3 (Figure 6). After invasion by Glu, Asp may have occupied sectors 4A, 3A, 2A, and 1A (Figure 7). Ultimately, Asp retained the most favored row 4A, abandoning all other sectors in column 3 (Figure 9). Glu retained favored row 4B and abandoned less favored rows 3B, 2B, and 1B to invading amino acids. Asn invaded row 3A. Lys invaded row 3B. Gln invaded row 2B. His invaded row 2A. Tyr and stop codons were late entries into the code occupying disfavored row 1 (3rd anticodon position A).

As noted above, we posit that Archaea are most similar to LUCA for translation functions [1,2,13,22]. For instance, archaeal tRNAs are more ancient than bacterial tRNAs. Therefore, to understand the initial evolution of the code requires a focus on archaeal systems. Notably, in Archaea, AspRS-IIB, AsnRS-IIB, and HisRS-IIA enzymes are reasonably closely related in structure and sequence (Figure 3A). Furthermore, in Archaea, GluRS-IB, LysRS-IC and GlnRS-IB are closely related enzymes in structure and sequence (Figure 3A). Evolution of aaRS enzymes, therefore, strongly supports our proposed model for column 3 evolution. Interestingly, in Bacteria, LysRS-IIB is closely related to AspRS-IIB and AsnRS-IIB in structure and sequence. Therefore, we posit that LysRS-IIB in Bacteria was evolved from AspRS-IIB, within column 3, reinforcing the importance of evolution within columns. The switch from LysRS-IC (Archaea) to LysRS-IIB (Bacteria) likely occurred close to the root of the archaeal→bacterial divergence. Clearly, evolution of the genetic code within column 3 (tRNA anticodon position 2 U) is demonstrated.

### 3.16. Evolution within Genetic Code Columns 

So, why did the genetic code evolve so clearly along code columns? The obvious answer is that the code initially evolved around the tRNA anticodon 2nd position, which is most important for translational accuracy. Assuming this idea is correct, what is the mechanism? One mechanism is indicated by translation systems in Archaea (and some Bacteria). We identify ancient Archaea, those most closely related to LUCA, as species with the least radiated tRNAomes from tRNA^Pri^ [1,3,14]. From analysis of tRNAomes, examples of ancient archaeal families include *Pyrococcus*, *Pyrobaculum*, *Staphylothermus*, *Aeropyrum,* and *Sulfolobus*. Ancient Archaea lack GlnRS-IIB. In place of GlnRS-IIB, these species charge tRNA^Gln^ with Glu using GluRS-IIB. Then a Glu-tRNA^Gln^ amidotransferase converts the complex to Gln-tRNA^Gln^ [66,67]. As described above, Met also invaded an Ile sector leading to the sharing of the CAU anticodon. Similar mechanisms appear to be common in sectoring the code within columns. A similar mechanism could potentially explain transitions leading to the occupation of the following code sectors: (1) Val→Leu; (2) Val→Ile; (3) Ile→Met; (4) Ala→Pro; (5) Ala→Thr; (6) Pro→Ser; (7) Asp→Asn; (8) Asp→His; and (9) Glu→Gln. In some cases, mischarging of tRNAs may simply occur by invasion of an amino acid from outside the code, as with Ile→Met, rather than metabolic modification of a charged tRNA, as with Glu→Gln and, we assume, Asp→Asn. Some of these sectoring events require additional steps, i.e., exchanging AlaRS-IIA with AlaRS-IID, a step that most likely occurred about the time of full sectoring of genetic code column 2. These posited events are best indicated from evolution of aaRS enzymes, which are often closely related within columns (Figure 3). To a lesser extent, these events may be indicated by analysis of tRNAomes, particularly in ancient Archaea [1,3]. tRNAs that evolve within genetic code columns tend to mutate and/or accumulate modifications rapidly to establish their new identity and to prevent mischarging. In columns 1 and 2, aaRS editing also suppresses mischarging of tRNAs.

### 3.17. An Alternate Model for Sectoring of Column 2

Because there are some limits to our clairvoyance describing events from ~4 billion years ago, we posit an alternate model for sectoring of column 2 and for Ser jumping from column 2 to column 4. Column 2 may have initially split between Ala (8-codon sector; rows 3 and 4) and Ser (8-codon sector; rows 1 and 2). After evolution of the EF-Tu latch, Ser may then have invaded the Ala sector, resulting in Ser occupying column 2, rows 1, 2, and 3, and Ala retaining favored row 4. Ser (GGU) may then have jumped to Ser (GCU) (column 4) via a single base change in tRNA^Ser^ (anticodon position 2). Subsequently, Pro invaded row 2, displacing Ser, and Thr invaded row 3, displacing Ser. Such a model has potential advantages over that described above. Notably, Ser jumping to favored column 4 is more easily described. Also, because of amino acid similarity, Ser→Thr appears to be a reasonable transition in row 3. Pro would displace Ser in row 2, leaving Ser in disfavored row 1, but this might not pose a difficulty if Ser had previously occupied favored column 4 row 3A. We give this example of an alternate model as a guide for understanding evolution of the genetic code. The model we advance is a working model. The detail in the model we find to be an advantage. Alternate pathways make slightly different predictions that potentially can be challenged computationally and/or by experiment. We know of no other model for code evolution that could potentially make such detailed or informative predictions.

### 3.18. An Alternate Model for Evolution of Stop Codons

A appears to be strongly disfavored in the 3rd anticodon position. We, therefore, posit that row 1 of the genetic code may not have initially been populated with amino acids. Instead, row 1 may have represented stop codons, and evolution of the EF-Tu latch may have been required to encode amino acids in row 1. In such a modified model, row 1 would occupy with amino acids only at the ~16 amino acid stage. This suggestion is a minor possible adjustment to the model described above. 

### 3.19. aaRS Editing

aaRS editing is a feature of some aaRS enzymes [56]. Editing allows a mischarged amino acid to be hydrolyzed from the tRNA 3’-end, using a separate active site from the aminoacylating active site and limiting amino acid misincorporation on the ribosome. Remarkably, in Archaea, aaRS enzymes that edit locate to columns 1 and 2 of the genetic code (Figure 2 and Figure 3). A partial exception is SerRS-IIA, which resides in column 4 as well as in column 2. As described above, it appears that Ser jumped from column 2 to column 4. In Archaea, ProRS-IIA does not edit, but, in Bacteria, ProRS-IIA does edit. The amino acids specified in columns 1 and 2 are hydrophobic (column 1) and neutral (column 2). Neutral amino acids in column 2 also have limited hydrogen-bonding capacity (i.e., Ser and Thr side chains are mostly limited to a single H-bond to an aaRS side chain). Editing is more essential for aaRS enzymes that have a decreased capacity to discriminate the amino acid substrate within their active sites. Editing, therefore, is most important for amino acids that are hydrophobic, neutral and/or have reduced H-bonding capacity (columns 1 and 2). The pattern of aaRS editing demonstrates additional unexpected structure in the genetic code.

### 3.20. Late Additions to the Genetic Code

Phe, Tyr, Trp, Cys, Met, and His are posited to be among the last additions to the genetic code [19,20,68]. Stop codons, which are read by protein release factors rather than tRNAs, are also posited to be late additions to the code. Phe, Tyr, Trp, and Cys are all in disfavored row 1 of the code (3rd anticodon position A). Stop codons, which do not follow tRNA anticodon rules [21], are in disfavored row 1 of the code. Met appears to have invaded the Ile sector as a late event. For tRNA^Met^ (CAU) and tRNA^Ile^ (CAU), wobble C covalent modifications, particularly to tRNA^Ile^ (agmatidine in Archaea; lysidine in Bacteria), are very important to discriminate tRNA^Met^ and tRNA^Ile^. tRNA^Met^ acceptor stems (As) are important to discriminate elongator tRNA^Met^ (i.e., 5’-As GCCCGGG) from initiator tRNA^Met^ (i.e., 5’-As AGCGGGA). In Archaea, tRNA^Ile^ (CAU) (C→agmatidine) is commonly used to recognize AUA Ile codons without recognition of AUG Met codons. The tRNA^Ile^ (CAU) C~A wobble pair is recognized by modification of the C to agmatidine to achieve this discrimination [53,54,55]. Only in eukaryotes is tRNA^Ile^ (UAU) commonly utilized. In eukaryotes, tRNA^Ile^ (IAU; I for inosine) can recognize the three Ile codons (AUC, AUU, AUA).

### 3.21. Alternate Genetic Code Models 

Focusing on tight coevolution of metabolic systems to describe patterns of genetic code evolution is likely a mistake. We posit that apparent parallels between genetic code evolution and amino acid metabolism evolution (i.e., evolution of the genetic code within columns) occur primarily because of evolution of the genetic code according to the tRNA anticodon, as described above. If the tRNA anticodon is the central feature determining the history of evolution of the genetic code, the entire scenario makes sense according to Darwinian principles. Otherwise, the sectoring of the code makes little to no sense. That stated, we do accept that coevolution of metabolism and the genetic code occurred. In some cases, modification of amino acids on tRNAs is a mechanism for code sectoring (i.e., Asp→Asn and Glu→Gln). In other cases, amino acids appear to invade from outside the code (i.e., Ile→Met). We argue that, with invasions from outside the code, coevolution of metabolism and the code provided the amino acids that were available to invade the code rather than metabolism driving the sectoring of the code.

Another potential error that we perceive in genetic code models is to build up the code a sector at a time. We do not believe such models are reasonable. Initially, we made the assumption that the entire code (all anticodons and all codons) was populated with tRNA^Gly^ (Figure 5). To our surprise, this simple assumption allowed a rich and highly detailed model for genetic code evolution with clear selection strategies to emerge. We were surprised because we commenced this effort thinking that, after ~4 billion years of evolution, development of such a detailed model was not possible. In support of our assumption, however, the tRNA anticodon is successfully mutated more rapidly than other tRNA sequences, which are required to support RNA stems and to stabilize tRNA structure. This is because the anticodon stem and loop sticks out from the tRNA. Other anticodon loop bases (7-nt loop positions 1, 2, 6, and 7) are highly constrained in sequence to maintain U-turn geometry, which is necessary to present a 3-nt anticodon. We imagined that tRNA initially was essential genetic evolutionary property (Figure 1). That is to say that tRNA was of value to synthesize polyglycine to stabilize protocells. We imagined that, being of value, tRNA was replicated rapidly in an RNA world, providing many copies for modification and genetic alteration. The other advantage to our model was that the model brought mRNA in line with tRNA, because, initially, all codons encoded glycine. In this way, tRNA anticodons and mRNA codons could more easily coevolve to encode incoming amino acids. In other scenarios, anticodons and codons must converge to encode products, which is more difficult to imagine. As anticodons evolved within the code, mRNA codons coevolved to encode ever more complex products. By contrast, a GC→GCA→GCAU genetic code (based on codons rather than anticodons) [52] evolutionary model does not adequately address this difficulty. Such a model begins within a sector and evolves to occupy other sectors. Such models have the critical flaw described above.

Another scheme for evolution of the genetic code is the GNC→SNS→standard code model (N = any base; S = G or C) [6]. We find some agreement and some disagreement with this model. One objection that we would raise is that the scheme is presented as a codon-centric model rather than an anticodon-centric model. Working out from tRNA is a big advantage, as we hope we have shown. Also, why begin with codon GNC (also anticodon GNC) rather than, for instance, CNG? Anticodon GNC gives Gly, Asp, Ala, and Val as the first four amino acids, which agrees with our assessment (Figure 4 and Figure 6). GNC also supports 3rd anticodon position C, which we deem favorable because of the C > G > U >> A anticodon rule. However, initial selection of GNC rather than CNG appears arbitrary. SNS makes some sense because C/G should facilitate 1st and 3rd position anticodon reading before evolution of the EF-Tu anticodon-codon latch. The SNS phase brings Gly, Asp, Ala, Val, Leu, Pro, Glu, Gln, His, and Arg into the code, which is very similar to the recruitment order we favor. Possible limitations of the alternate GNC model include: (1) apparent favoring of codons over anticodons; (2) beginning the code arbitrarily with GNC, rather than SNS; (3) no consideration of the EF-Tu latch in anticodon-codon reading; (4) building up the code via sectors rather than filling in and invading; (5) awkward co-evolution of anticodons and codons; and (6) little consideration of why 4- and 2-codon sectors are favored over 1-codon sectors. A recent paper on this subject [6] utilizes an incorrect tRNA evolution model and an incorrect aaRS evolution model. The model does not fully recognize the extent to which aaRS enzyme evolution parallels the establishment of the code (Figure 3).

### 3.22. Alternate Representations of the Genetic Code

We strongly advocate for the representation of the genetic code shown in Figure 2 and Figure 3. Many other representations of the genetic code are possible. Circular representations of the code emphasize selection for G = C pairs in anticodon-codon interactions [69]. As we argue here, however, the Darwinian selection for the tRNA anticodon positions 2 and 3 appears to be C > G > U >> A, rather than for both C and G to be strongly favored in the anticodon. We reject codon tables lacking anticodons because the genetic code evolved around the tRNA anticodon. Recognizing the central importance of tRNA and the tRNA anticodon, as we do here, provides remarkable insight into genetic code evolution.

## 4. Life on Another Planet

If life were to evolve independently on a planet or moon away from Earth, how would organisms evolve coding? Would such organisms evolve tRNA or a tRNA-like coding molecule? Would the coding molecule be RNA? It is very difficult to imagine an alternate scheme for chemical coding of comparable complexity to tRNA-based coding. It is also difficult to evolve a more complex code than the standard 3-nt genetic code. Notably, tRNA has a perfectly structured RNA anticodon loop to support a 3-nt code. The anticodon loop is 7-nt. Significantly, a 6- or 8-nt loop cannot support a compact U-turn geometry or as tight a loop. A tight anticodon loop is necessary to support anticodon-codon interactions during translation. So, with RNA as the genetic material, only a 3-nt code appears reasonable. We recognize that 4-nt codes are conceivable and demonstrated by engineering with tRNA, but (to our knowledge) 4-nt codes are not observed in free-living organisms. To increase the coding potential of the standard genetic code would require scrambling the standard code. For instance, 4-codon sectors on the left half of the code might be split into 2-codon sectors, to add amino acids, but to avoid error catastrophe, hydrophobic amino acids would have to share sectors with amino acids with more distinct character (i.e., charge and/or H-bonding capacity). So, coding capacity might be increased by aggressive engineering, but evolving a much more complex code than the standard code under conditions of natural selection appears unlikely. At most, only a few additional amino acids could be added to the naturally evolved code. The 21-assignment standard code is very close to the theoretical 32-assignment limit.

So, tRNA is a highly specialized molecule constructed from ligation of two different types of 31-nt minihelix (1-D loop minihelix + 2-anticodon loop/T loop minihelices) [1,2,4]. Very likely, tRNA could not be constructed of three identical minihelices (i.e., 3-anticodon loop/T loop minihelices). During pre-life, such a molecule would probably be processed into 3 separate 31-nt minihelices by then-existing ribozyme ribo-endonucleases. From the tRNA structure, it is likely that neither the anticodon loop nor the T loop minihelix could be substituted with another minihelix. For instance, as the anticodon loop, a D loop minihelix would likely support a 1-nt rather than a 3-nt code. Significantly, the D loop minihelix cannot support a 7-nt U-turn loop. It is very likely that the D loop minihelix component of tRNA could be re-engineered with a different sequence to form a tRNA-like molecule that might serve for coding. Such a tRNA variant might support coding and life but would be highly analogous to tRNA on Earth and would support a similar code with similar coding capacity. The authors of this paper cannot yet imagine a chemical tRNA substitute with advantages over the tRNA found on Earth, to support or engineer a richer coding system.

## 5. Predictions

The 3-minihelix model we propose for evolution of tRNA makes many predictions, most of which are confirmed [4]. We proposed that the anticodon loop and the T loop are homologs and then showed that they are [1,2,4]. We proposed that the D loop minihelix core was initially a UAGCC repeat and then showed that this prediction was confirmed [2]. We proposed that the last 5 nt of the D loop and the V loop were derived from acceptor stems, and we confirmed this prediction [2,4,13]. We proposed that acceptor stems were based on a GCG repeat and its CGC complement. We confirmed this prediction [2,4]. We posited that the expanded V loop in type II tRNA was initially a 3’-acceptor stem ligated to a 5’-acceptor stem and then showed that this was the case [13]. In ancient Archaea (i.e., *Pyrococcus furiosis*), tRNA^Pri^ (the primordial tRNA) is almost identical to tRNA^Gly^, indicating that Gly was the first encoded amino acid. We have shown that no 2-minihelix model [23,26,27,70,71,72] is adequate to describe tRNA evolution, because (for instance) such models are inconsistent with obvious anticodon loop and T loop homology [1,2,4,13]. Only a 3-minihelix model is adequate to describe tRNA evolution. No accretion model, in which tRNA grows a stem at a time, is adequate to describe tRNA evolution because accretion models are inconsistent with (for instance) anticodon loop and T loop homology. 

Based on tRNA evolution models and our analysis of aaRS evolution, we propose a working model for evolution of the genetic code [1]. This model also makes multiple predictions, some of which are already tested. Our model predicts that tRNAomes in ancient organisms are more highly ordered than tRNAomes in more derived organisms, as we have shown [1,3,13]. We predict that a telomerase-like ribozyme can be generated to form RNA repeats (i.e., GCG, CGC, and UAGCC) (Figure 1). We predict many ribozymes that have previously been generated in vitro [34,36,37,39,40,42,73,74]. We predict a peptidyl transferase ribozyme, which has been generated [34]. We predict that tRNAomes evolve both by divergence and convergence within a limited sequence space. This idea has been partly tested. We predict that Archaea is more similar to LUCA than Bacteria for transcription and translation functions [1,2,3,8,9,10,13,22]. This model is strongly supported in multiple ways. We predict the standard code (20 amino acids + stops) is approximately the most complex code that could easily evolve and that the maximum code complexity (32-assignments) can be approached, but not achieved, because of fidelity mechanisms [1,3,13,14]. We predicted evolution of the genetic code within columns (2nd anticodon position) and we demonstrate this (Figure 2 and Figure 3). We predicted that the genetic code has order based on its evolutionary history, which we demonstrate.

## 6. The Frozen Accident

The standard genetic code evolved quickly (within ~300 million years; Figure 1), and, once formed, did not change very much thereafter. Evidence for this conclusion is the obvious conservation of genetic code evolution patterns reflected in patterns of aaRS evolution (i.e., Figure 3). Innovation in forming the code was driven by tRNA charging modifications and errors [1,3,13,14]. Alteration of Glu-tRNA^Gln^ to Gln-tRNA^Gln^ by a Glu-tRNA^Gln^ amidotransferase is an example of an intermediate in sectoring the genetic code to encode both Glu and Gln [66,67,75]. Subsequently, a GlnRS can evolve to supplant the Glu-tRNA^Gln^ amidotransferase. Met invading an Ile 4-codon sector reflects another mechanism to enrich the code [53,54,55]. In this case, the sectoring was never completed. In the case of Met invading Ile, Met appears to begin incorporation by mischarging tRNA^Ile^ to Met-tRNA^Ile^. Then a duplicated tRNA^Ile^ (CAU) mutates to tRNA^Met^ (CAU), and IleRS duplicates, and one copy evolves to MetRS. So, tRNA charging errors and modifications of amino acids bound to tRNAs drove innovation of the code. Evolving translational fidelity mechanisms, therefore, drives the code toward closure by inhibiting further amino acid additions. Fidelity mechanisms include aaRS editing, evolution of the aaRS synthetic site (to enhance accuracy of amino acid addition), the EF-Tu latch, tRNA modifications, tRNA evolution, and amino acid character (i.e., size, H-bonding, charge, hydrophobicity). It appears, for instance, that aaRS editing in columns 1 and 2 of the code helped to protect 4-codon sectors from division to two 2-codon sectors, limiting the complexity of the code [1,14].

## References

[1] Kim Y., Opron K., Burton Z.F. (2019). A tRNA- and Anticodon-Centric View of the Evolution of Aminoacyl-tRNA Synthetases, tRNAomes, and the Genetic Code. Life.

[2] Pak D., Root-Bernstein R., Burton Z.F. (2017). tRNA structure and evolution and standardization to the three nucleotide genetic code. Transcription.

[3] Pak D., Du N., Kim Y., Sun Y., Burton Z.F. (2018). Rooted tRNAomes and evolution of the genetic code. Transcription.

[4] Burton Z.F. (2020). The 3-Minihelix tRNA Evolution Theorem. J. Mol. Evol..

[5] Mariscal C., Barahona A., Aubert-Kato N., Aydinoglu A.U., Bartlett S., Cardenas M.L., Chandru K., Cleland C., Cocanougher B.T., Comfort N. (2019). Hidden Concepts in the History and Philosophy of Origins-of-Life Studies: A Workshop Report. Orig. Life Evol. Biosph..

[6] Chatterjee S., Yadav S. (2019). The Origin of Prebiotic Information System in the Peptide/RNA World: A Simulation Model of the Evolution of Translation and the Genetic Code. Life.

[7] Opron K., Burton Z.F. (2019). Ribosome Structure, Function, and Early Evolution. Int. J. Mol. Sci..

[8] Burton Z.F., Opron K., Wei G., Geiger J.H. (2016). A model for genesis of transcription systems. Transcription.

[9] Burton Z.F. (2014). The Old and New Testaments of gene regulation. Evolution of multi-subunit RNA polymerases and co-evolution of eukaryote complexity with the RNAP II CTD. Transcription.

[10] Burton S.P., Burton Z.F. (2014). The sigma enigma: Bacterial sigma factors, archaeal TFB and eukaryotic TFIIB are homologs. Transcription.

[11] Ma W. (2017). What Does “the RNA World” Mean to “the Origin of Life”?. Life.

[12] Kunnev D., Gospodinov A. (2018). Possible Emergence of Sequence Specific RNA Aminoacylation via Peptide Intermediary to Initiate Darwinian Evolution and Code Through Origin of Life. Life.

[13] Kim Y., Kowiatek B., Opron K., Burton Z.F. (2018). Type-II tRNAs and Evolution of Translation Systems and the Genetic Code. Int. J. Mol. Sci..

[14] Pak D., Kim Y., Burton Z.F. (2018). Aminoacyl-tRNA synthetase evolution and sectoring of the genetic code. Transcription.

[15] Saint-Leger A., Bello C., Dans P.D., Torres A.G., Novoa E.M., Camacho N., Orozco M., Kondrashov F.A., Ribas de Pouplana L. (2016). Saturation of recognition elements blocks evolution of new tRNA identities. Sci. Adv..

[16] Bernhardt H.S. (2016). Clues to tRNA Evolution from the Distribution of Class II tRNAs and Serine Codons in the Genetic Code. Life.

[17] Bernhardt H.S., Patrick W.M. (2014). Genetic code evolution started with the incorporation of glycine, followed by other small hydrophilic amino acids. J. Mol. Evol..

[18] Bernhardt H.S., Tate W.P. (2008). Evidence from glycine transfer RNA of a frozen accident at the dawn of the genetic code. Biol. Direct.

[19] Brooks D.J., Fresco J.R., Lesk A.M., Singh M. (2002). Evolution of amino acid frequencies in proteins over deep time: Inferred order of introduction of amino acids into the genetic code. Mol. Biol. Evol..

[20] Fournier G.P., Alm E.J. (2015). Ancestral Reconstruction of a Pre-LUCA Aminoacyl-tRNA Synthetase Ancestor Supports the Late Addition of Trp to the Genetic Code. J. Mol. Evol..

[21] Burroughs A.M., Aravind L. (2019). The Origin and Evolution of Release Factors: Implications for Translation Termination, Ribosome Rescue, and Quality Control Pathways. Int. J. Mol. Sci..

[22] Battistuzzi F.U., Feijao A., Hedges S.B. (2004). A genomic timescale of prokaryote evolution: Insights into the origin of methanogenesis, phototrophy, and the colonization of land. BMC Evol. Biol..

[23] Di Giulio M. (2019). A comparison between two models for understanding the origin of the tRNA molecule. J. Theor. Biol..

[24] Demongeot J., Seligmann H. (2020). RNA Rings Strengthen Hairpin Accretion Hypotheses for tRNA Evolution: A Reply to Commentaries by Z.F. Burton and M. Di Giulio. J. Mol. Evol..

[25] Di Giulio M. (2020). An RNA Ring was Not the Progenitor of the tRNA Molecule. J. Mol. Evol..

[26] Di Giulio M. (2012). The origin of the tRNA molecule: Independent data favor a specific model of its evolution. Biochimie.

[27] Branciamore S., Di Giulio M. (2011). The presence in tRNA molecule sequences of the double hairpin, an evolutionary stage through which the origin of this molecule is thought to have passed. J. Mol. Evol..

[28] Demongeot J., Seligmann H. (2019). The Uroboros Theory of Life’s Origin: 22-Nucleotide Theoretical Minimal RNA Rings Reflect Evolution of Genetic Code and tRNA-rRNA Translation Machineries. Acta Biotheor..

[29] Demongeot J., Glade N., Moreira A., Vial L. (2009). RNA relics and origin of life. Int. J. Mol. Sci..

[30] Demongeot J., Moreira A. (2007). A possible circular RNA at the origin of life. J. Theor. Biol..

[31] Pinho M.G., Kjos M., Veening J.W. (2013). How to get (a)round: Mechanisms controlling growth and division of coccoid bacteria. Nat. Rev. Microbiol..

[32] Zapun A., Vernet T., Pinho M.G. (2008). The different shapes of cocci. FEMS Microbiol. Rev..

[33] Scheffers D.J., Pinho M.G. (2005). Bacterial cell wall synthesis: New insights from localization studies. Microbiol. Mol. Biol. Rev..

[34] Zhang B., Cech T.R. (1998). Peptidyl-transferase ribozymes: Trans reactions, structural characterization and ribosomal RNA-like features. Chem. Biol..

[35] Pressman A.D., Liu Z., Janzen E., Blanco C., Muller U.F., Joyce G.F., Pascal R., Chen I.A. (2019). Mapping a Systematic Ribozyme Fitness Landscape Reveals a Frustrated Evolutionary Network for Self-Aminoacylating RNA. J. Am. Chem. Soc..

[36] Illangasekare M., Yarus M. (2012). Small aminoacyl transfer centers at GU within a larger RNA. RNA Biol..

[37] Yarus M. (2011). The meaning of a minuscule ribozyme. Philos. Trans. R Soc. Lond. B Biol. Sci..

[38] Turk R.M., Illangasekare M., Yarus M. (2011). Catalyzed and spontaneous reactions on ribozyme ribose. J. Am. Chem. Soc..

[39] Turk R.M., Chumachenko N.V., Yarus M. (2010). Multiple translational products from a five-nucleotide ribozyme. Proc. Natl. Acad. Sci. USA.

[40] Chumachenko N.V., Novikov Y., Yarus M. (2009). Rapid and simple ribozymic aminoacylation using three conserved nucleotides. J. Am. Chem. Soc..

[41] Kim D.E., Joyce G.F. (2004). Cross-catalytic replication of an RNA ligase ribozyme. Chem. Biol..

[42] Paul N., Joyce G.F. (2002). A self-replicating ligase ribozyme. Proc. Natl. Acad. Sci. USA.

[43] Rogers J., Joyce G.F. (2001). The effect of cytidine on the structure and function of an RNA ligase ribozyme. RNA.

[44] Jaeger L., Wright M.C., Joyce G.F. (1999). A complex ligase ribozyme evolved in vitro from a group I ribozyme domain. Proc. Natl. Acad. Sci. USA.

[45] Koonin E.V. (2017). Frozen Accident Pushing 50: Stereochemistry, Expansion, and Chance in the Evolution of the Genetic Code. Life.

[46] Koonin E.V., Novozhilov A.S. (2017). Origin and Evolution of the Universal Genetic Code. Annu. Rev. Genet..

[47] Koonin E.V., Novozhilov A.S. (2009). Origin and evolution of the genetic code: The universal enigma. IUBMB Life.

[48] Caetano-Anolles D., Caetano-Anolles G. (2016). Piecemeal Buildup of the Genetic Code, Ribosomes, and Genomes from Primordial tRNA Building Blocks. Life.

[49] Rogers S.O. (2019). Evolution of the genetic code based on conservative changes of codons, amino acids, and aminoacyl tRNA synthetases. J. Theor. Biol..

[50] Barbieri M. (2019). Evolution of the genetic code: The ambiguity-reduction theory. Biosystems.

[51] Barbieri M. (2019). A general model on the origin of biological codes. Biosystems.

[52] Hartman H., Smith T.F. (2019). Origin of the Genetic Code Is Found at the Transition between a Thioester World of Peptides and the Phosphoester World of Polynucleotides. Life.

[53] Satpati P., Bauer P., Aqvist J. (2014). Energetic tuning by tRNA modifications ensures correct decoding of isoleucine and methionine on the ribosome. Chemistry.

[54] Voorhees R.M., Mandal D., Neubauer C., Kohrer C., RajBhandary U.L., Ramakrishnan V. (2013). The structural basis for specific decoding of AUA by isoleucine tRNA on the ribosome. Nat. Struct. Mol. Biol..

[55] Mandal D., Kohrer C., Su D., Russell S.P., Krivos K., Castleberry C.M., Blum P., Limbach P.A., Soll D., RajBhandary U.L. (2010). Agmatidine, a modified cytidine in the anticodon of archaeal tRNA(Ile), base pairs with adenosine but not with guanosine. Proc. Natl. Acad. Sci. USA.

[56] Perona J.J., Gruic-Sovulj I. (2014). Synthetic and editing mechanisms of aminoacyl-tRNA synthetases. Top. Curr. Chem..

[57] Kun A., Radvanyi A. (2018). The evolution of the genetic code: Impasses and challenges. Biosystems.

[58] Ribas de Pouplana L., Torres A.G., Rafels-Ybern A. (2017). What Froze the Genetic Code?. Life.

[59] Doig A.J. (2017). Frozen, but no accident—Why the 20 standard amino acids were selected. FEBS J..

[60] Loveland A.B., Demo G., Grigorieff N., Korostelev A.A. (2017). Ensemble cryo-EM elucidates the mechanism of translation fidelity. Nature.

[61] Rozov A., Wolff P., Grosjean H., Yusupov M., Yusupova G., Westhof E. (2018). Tautomeric G*U pairs within the molecular ribosomal grip and fidelity of decoding in bacteria. Nucleic Acids Res..

[62] Rozov A., Demeshkina N., Westhof E., Yusupov M., Yusupova G. (2016). New Structural Insights into Translational Miscoding. Trends Biochem. Sci..

[63] Rozov A., Westhof E., Yusupov M., Yusupova G. (2016). The ribosome prohibits the G*U wobble geometry at the first position of the codon-anticodon helix. Nucleic Acids Res..

[64] Blazej P., Wnetrzak M., Mackiewicz D., Mackiewicz P. (2018). Optimization of the standard genetic code according to three codon positions using an evolutionary algorithm. PLoS ONE.

[65] Blazej P., Wnetrzak M., Mackiewicz D., Gagat P., Mackiewicz P. (2019). Many alternative and theoretical genetic codes are more robust to amino acid replacements than the standard genetic code. J. Theor. Biol..

[66] Nureki O., O’Donoghue P., Watanabe N., Ohmori A., Oshikane H., Araiso Y., Sheppard K., Soll D., Ishitani R. (2010). Structure of an archaeal non-discriminating glutamyl-tRNA synthetase: A missing link in the evolution of Gln-tRNAGln formation. Nucleic Acids Res..

[67] Rampias T., Sheppard K., Soll D. (2010). The archaeal transamidosome for RNA-dependent glutamine biosynthesis. Nucleic Acids Res..

[68] Mukai T., Reynolds N.M., Crnkovic A., Soll D. (2017). Bioinformatic Analysis Reveals Archaeal tRNATyr and tRNATrp Identities in Bacteria. Life.

[69] Grosjean H., Westhof E. (2016). An integrated, structure- and energy-based view of the genetic code. Nucleic Acids Res..

[70] Widmann J., Di Giulio M., Yarus M., Knight R. (2005). tRNA creation by hairpin duplication. J. Mol. Evol..

[71] Nagaswamy U., Fox G.E. (2003). RNA ligation and the origin of tRNA. Orig. Life Evol. Biosph..

[72] Di Giulio I., McFadyen B.J., Blanchet S., Reeves N.D., Baltzopoulos V., Maganaris C.N. (2020). Mobile phone use impairs stair gait: A pilot study on young adults. Appl. Ergon..

[73] Horning D.P., Joyce G.F. (2016). Amplification of RNA by an RNA polymerase ribozyme. Proc. Natl. Acad. Sci. USA.

[74] McGinness K.E., Joyce G.F. (2003). In search of an RNA replicase ribozyme. Chem. Biol..

[75] O’Donoghue P., Sheppard K., Nureki O., Soll D. (2011). Rational design of an evolutionary precursor of glutaminyl-tRNA synthetase. Proc. Natl. Acad. Sci. USA.

